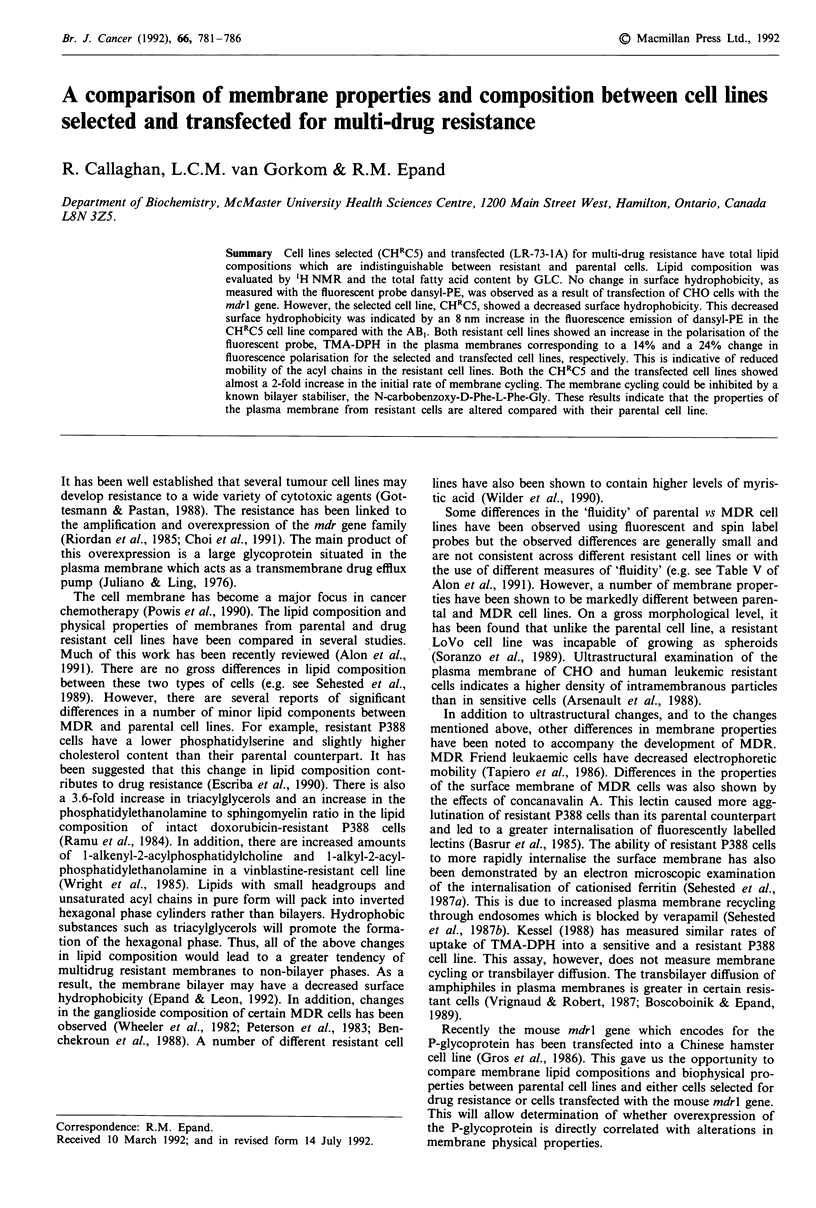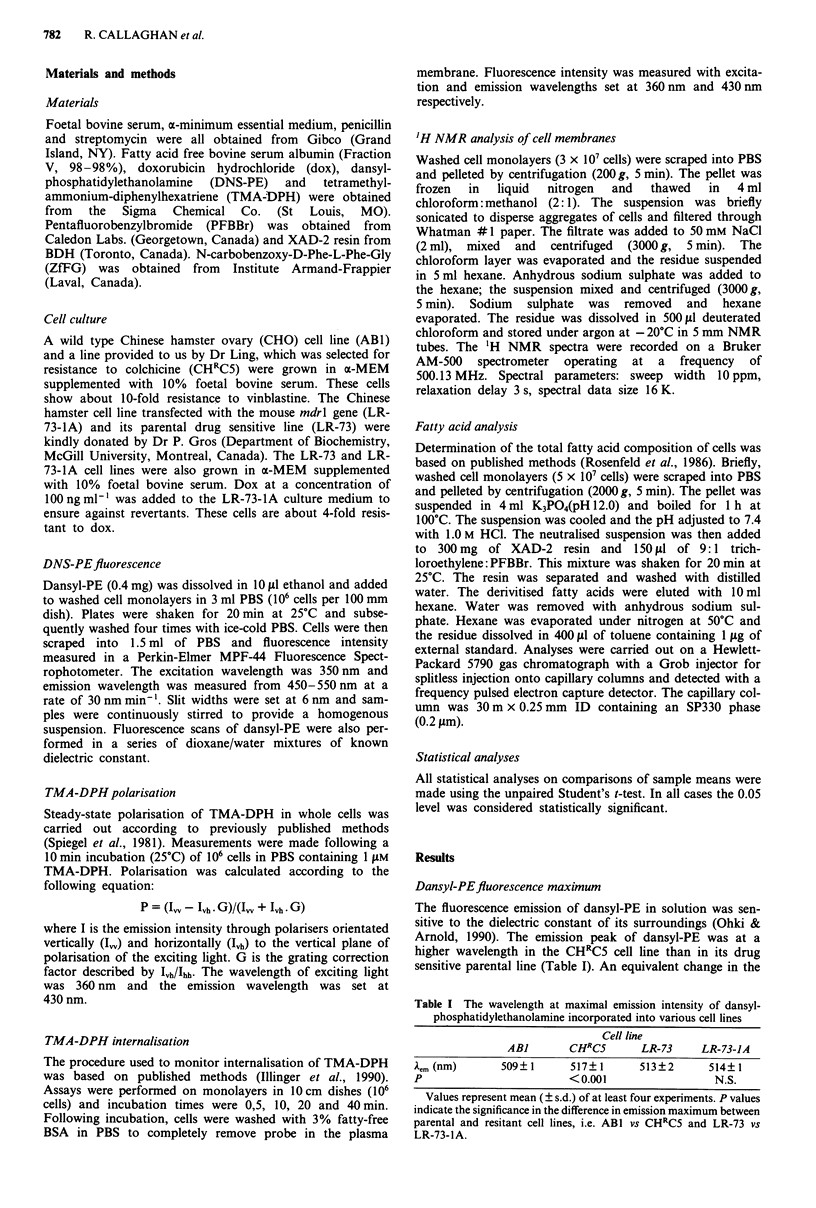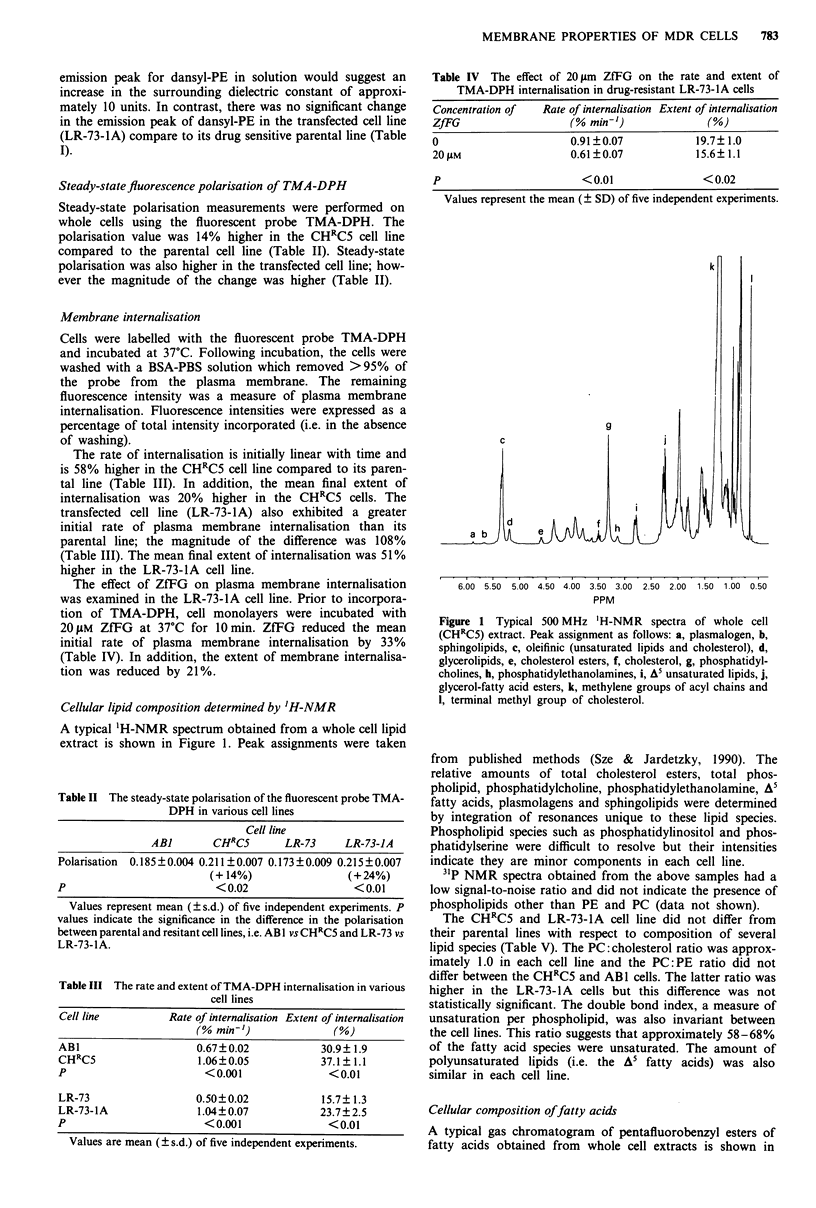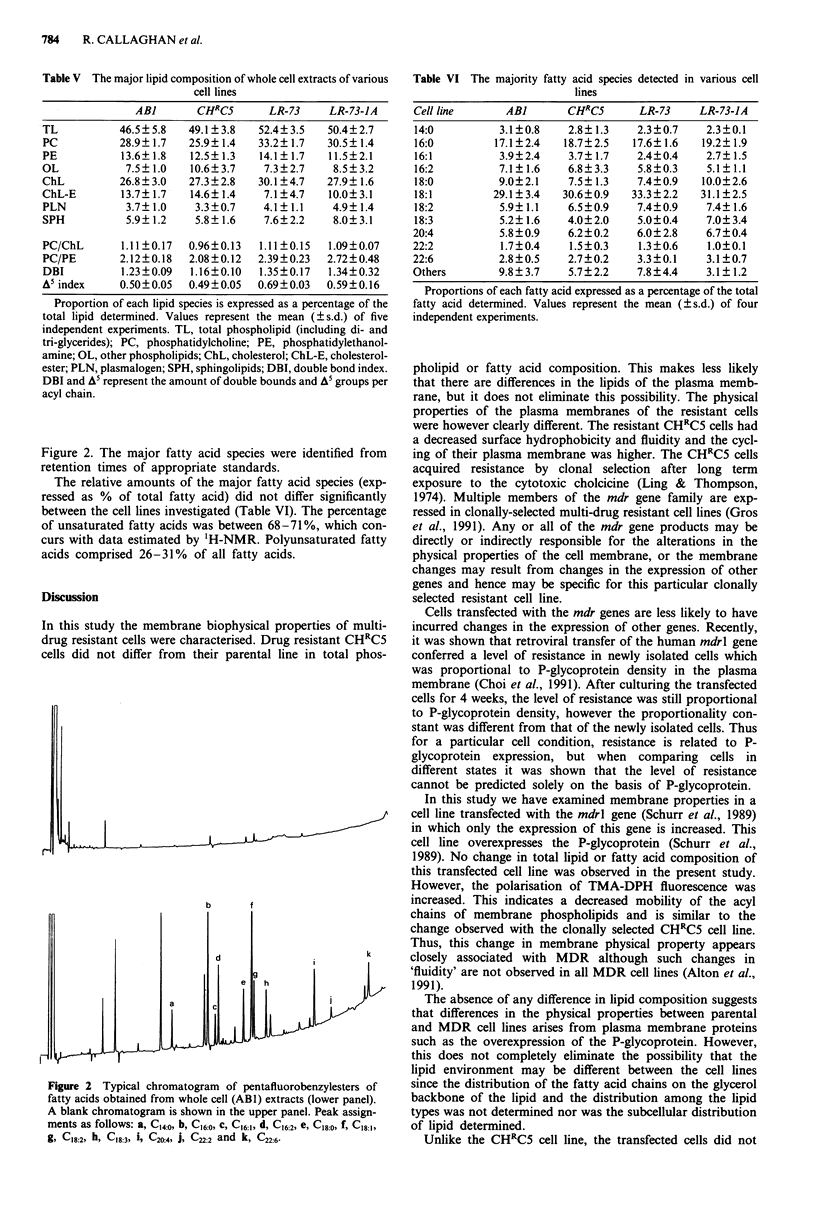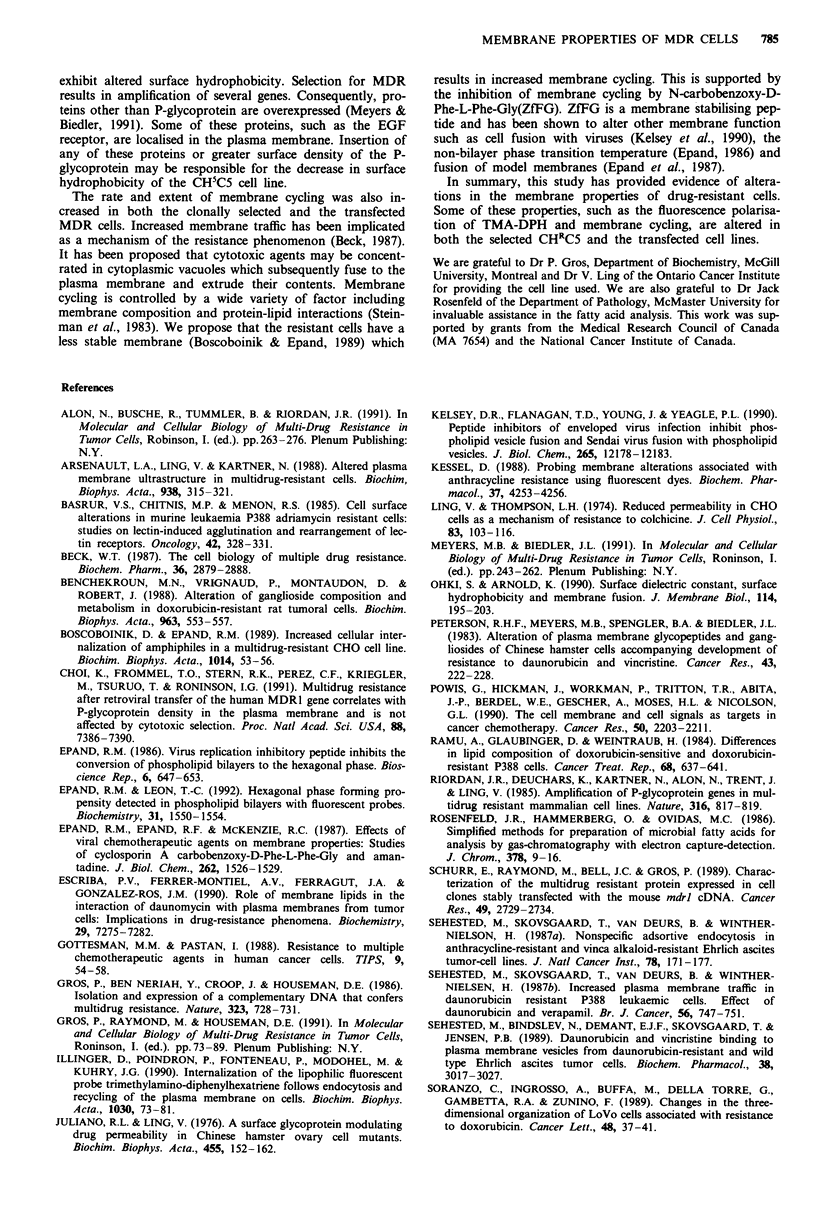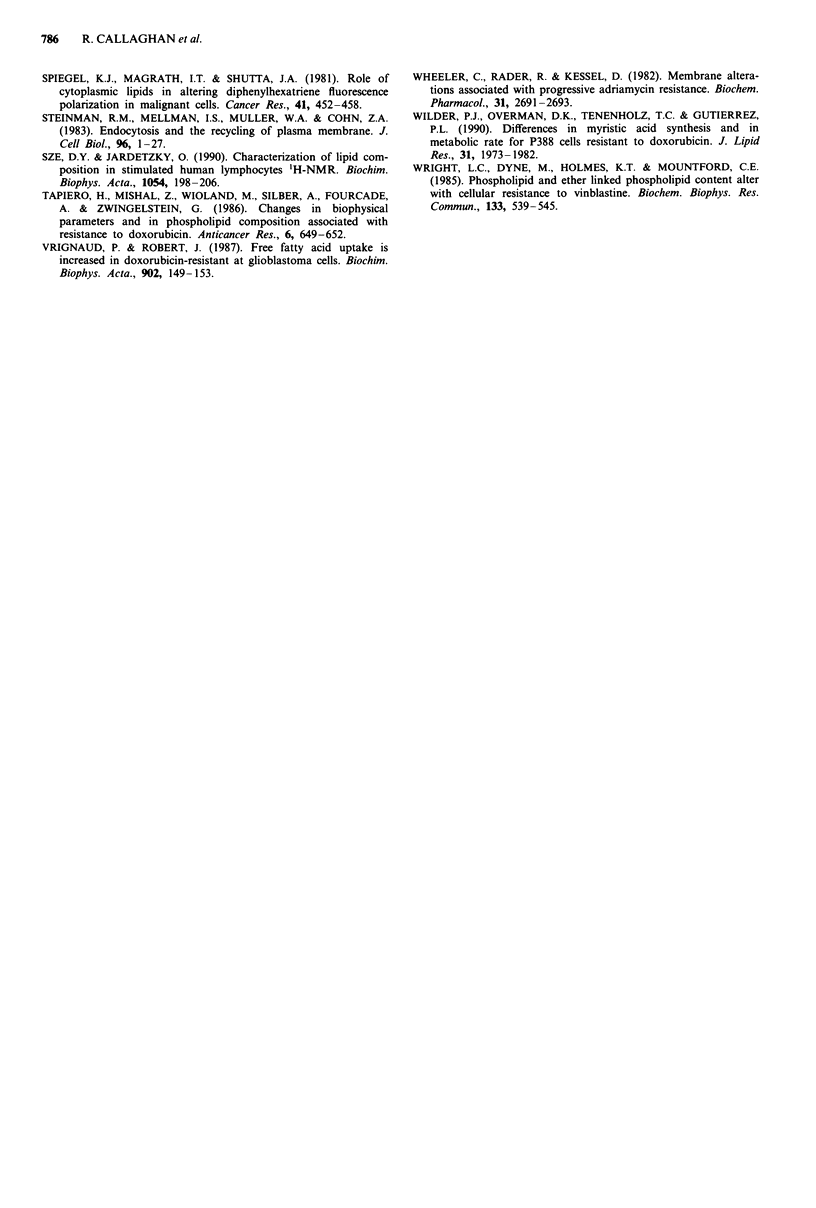# A comparison of membrane properties and composition between cell lines selected and transfected for multi-drug resistance.

**DOI:** 10.1038/bjc.1992.360

**Published:** 1992-11

**Authors:** R. Callaghan, L. C. van Gorkom, R. M. Epand

**Affiliations:** Department of Biochemistry, McMaster University Health Sciences Centre, Hamilton, Ontario, Canada.

## Abstract

Cell lines selected (CHRC5) and transfected (LR-73-1A) for multi-drug resistance have total lipid compositions which are indistinguishable between resistant and parental cells. Lipid composition was evaluated by 1H NMR and the total fatty acid content by GLC. No change in surface hydrophobicity, as measured with the fluorescent probe dansyl-PE, was observed as a result of transfection of CHO cells with the mdr1 gene. However, the selected cell line, CHRC5, showed a decreased surface hydrophobicity. This decreased surface hydrophobicity was indicated by an 8 nm increase in the fluorescence emission of dansyl-PE in the CHRC5 cell line compared with the AB1. Both resistant cell lines showed an increase in the polarisation of the fluorescent probe, TMA-DPH in the plasma membranes corresponding to a 14% and a 24% change in fluorescence polarisation for the selected and transfected cell lines, respectively. This is indicative of reduced mobility of the acyl chains in the resistant cell lines. Both the CHRC5 and the transfected cell lines showed almost a 2-fold increase in the initial rate of membrane cycling. The membrane cycling could be inhibited by a known bilayer stabiliser, the N-carbobenzoxy-D-Phe-L-Phe-Gly. These results indicate that the properties of the plasma membrane from resistant cells are altered compared with their parental cell line.


Br. J. Cancer (1992), **66**, 781–786 © Macmillan Press Ltd., 1992

### A comparison of membrane properties and composition between cell lines selected and transfected for multi-drug resistance

R. Callaghan, L.C.M. van Gorkom & R.M. Epand

*Department of Biochemistry, McMaster University Health Sciences Centre, 1200 Main Street West, Hamilton, Ontario, Canada L8N 3Z5.*

**Summary** Cell lines selected ($CH^RC5$) and transfected (LR-73-1A) for multi-drug resistance have total lipid compositions which are indistinguishable between resistant and parental cells. Lipid composition was evaluated by $^1$H NMR and the total fatty acid content by GLC. No change in surface hydrophobicity, as measured with the fluorescent probe dansyl-PE, was observed as a result of transfection of CHO cells with the *mdr*1 gene. However, the selected cell line, $CH^RC5$, showed a decreased surface hydrophobicity. This decreased surface hydrophobicity was indicated by an 8 nm increase in the fluorescence emission of dansyl-PE in the $CH^RC5$ cell line compared with the $AB_1$. Both resistant cell lines showed an increase in the polarisation of the fluorescent probe, TMA-DPH in the plasma membranes corresponding to a 14% and a 24% change in fluorescence polarisation for the selected and transfected cell lines, respectively. This is indicative of reduced mobility of the acyl chains in the resistant cell lines. Both the $CH^RC5$ and the transfected cell lines showed almost a 2-fold increase in the initial rate of membrane cycling. The membrane cycling could be inhibited by a known bilayer stabiliser, the N-carbobenzoxy-D-Phe-L-Phe-Gly. These results indicate that the properties of the plasma membrane from resistant cells are altered compared with their parental cell line.

It has been well established that several tumour cell lines may develop resistance to a wide variety of cytotoxic agents (Gottesmann & Pastan, 1988). The resistance has been linked to the amplification and overexpression of the *mdr* gene family (Riordan *et al.*, 1985; Choi *et al.*, 1991). The main product of this overexpression is a large glycoprotein situated in the plasma membrane which acts as a transmembrane drug efflux pump (Juliano & Ling, 1976).

The cell membrane has become a major focus in cancer chemotherapy (Powis *et al.*, 1990). The lipid composition and physical properties of membranes from parental and drug resistant cell lines have been compared in several studies. Much of this work has been recently reviewed (Alon *et al.*, 1991). There are no gross differences in lipid composition between these two types of cells (e.g. see Sehested *et al.*, 1989). However, there are several reports of significant differences in a number of minor lipid components between MDR and parental cell lines. For example, resistant P388 cells have a lower phosphatidylserine and slightly higher cholesterol content than their parental counterpart. It has been suggested that this change in lipid composition contributes to drug resistance (Escriba *et al.*, 1990). There is also a 3.6-fold increase in triacylglycerols and an increase in the phosphatidylethanolamine to sphingomyelin ratio in the lipid composition of intact doxorubicin-resistant P388 cells (Ramu *et al.*, 1984). In addition, there are increased amounts of 1-alkenyl-2-acylphosphatidylcholine and 1-alkyl-2-acylphosphatidylethanolamine in a vinblastine-resistant cell line (Wright *et al.*, 1985). Lipids with small headgroups and unsaturated acyl chains in pure form will pack into inverted hexagonal phase cylinders rather than bilayers. Hydrophobic substances such as triacylglycerols will promote the formation of the hexagonal phase. Thus, all of the above changes in lipid composition would lead to a greater tendency of multidrug resistant membranes to non-bilayer phases. As a result, the membrane bilayer may have a decreased surface hydrophobicity (Epand & Leon, 1992). In addition, changes in the ganglioside composition of certain MDR cells has been observed (Wheeler *et al.*, 1982; Peterson *et al.*, 1983; Benchekroun *et al.*, 1988). A number of different resistant cell lines have also been shown to contain higher levels of myristic acid (Wilder *et al.*, 1990).

Some differences in the 'fluidity' of parental *vs* MDR cell lines have been observed using fluorescent and spin label probes but the observed differences are generally small and are not consistent across different resistant cell lines or with the use of different measures of 'fluidity' (e.g. see Table V of Alon *et al.*, 1991). However, a number of membrane properties have been shown to be markedly different between parental and MDR cell lines. On a gross morphological level, it has been found that unlike the parental cell line, a resistant LoVo cell line was incapable of growing as spheroids (Soranzo *et al.*, 1989). Ultrastructural examination of the plasma membrane of CHO and human leukemic resistant cells indicates a higher density of intramembranous particles than in sensitive cells (Arsenault *et al.*, 1988).

In addition to ultrastructural changes, and to the changes mentioned above, other differences in membrane properties have been noted to accompany the development of MDR. MDR Friend leukaemic cells have decreased electrophoretic mobility (Tapiero *et al.*, 1986). Differences in the properties of the surface membrane of MDR cells was also shown by the effects of concanavalin A. This lectin caused more agglutination of resistant P388 cells than its parental counterpart and led to a greater internalisation of fluorescently labelled lectins (Basrur *et al.*, 1985). The ability of resistant P388 cells to more rapidly internalise the surface membrane has also been demonstrated by an electron microscopic examination of the internalisation of cationised ferritin (Sehested *et al.*, 1987*a*). This is due to increased plasma membrane recycling through endosomes which is blocked by verapamil (Sehested *et al.*, 1987*b*). Kessel (1988) has measured similar rates of uptake of TMA-DPH into a sensitive and a resistant P388 cell line. This assay, however, does not measure membrane cycling or transbilayer diffusion. The transbilayer diffusion of amphiphiles in plasma membranes is greater in certain resistant cells (Vrignaud & Robert, 1987; Boscoboinik & Epand, 1989).

Recently the mouse *mdr*1 gene which encodes for the P-glycoprotein has been transfected into a Chinese hamster cell line (Gros *et al.*, 1986). This gave us the opportunity to compare membrane lipid compositions and biophysical properties between parental cell lines and either cells selected for drug resistance or cells transfected with the mouse *mdr*1 gene. This will allow determination of whether overexpression of the P-glycoprotein is directly correlated with alterations in membrane physical properties.

Correspondence: R.M. Epand.
Received 10 March 1992; and in revised form 14 July 1992.

### Materials and methods

### Materials

Foetal bovine serum, α-minimum essential medium, penicillin and streptomycin were all obtained from Gibco (Grand Island, NY). Fatty acid free bovine serum albumin (Fraction V, 98–98%), doxorubicin hydrochloride (dox), dansyl-phosphatidylethanolamine (DNS-PE) and tetramethyl-ammonium-diphenylhexatriene (TMA-DPH) were obtained from the Sigma Chemical Co. (St Louis, MO). Pentafluorobenzylbromide (PFBBr) was obtained from Caledon Labs. (Georgetown, Canada) and XAD-2 resin from BDH (Toronto, Canada). N-carbobenzoxy-D-Phe-L-Phe-Gly (ZfFG) was obtained from Institute Armand-Frappier (Laval, Canada).

### Cell culture

A wild type Chinese hamster ovary (CHO) cell line (AB1) and a line provided to us by Dr Ling, which was selected for resistance to colchicine ($CH^RC5$) were grown in α-MEM supplemented with 10% foetal bovine serum. These cells show about 10-fold resistance to vinblastine. The Chinese hamster cell line transfected with the mouse *mdr*1 gene (LR-73-1A) and its parental drug sensitive line (LR-73) were kindly donated by Dr P. Gros (Department of Biochemistry, McGill University, Montreal, Canada). The LR-73 and LR-73-1A cell lines were also grown in α-MEM supplemented with 10% foetal bovine serum. Dox at a concentration of 100 ng $ml^{-1}$ was added to the LR-73-1A culture medium to ensure against revertants. These cells are about 4-fold resistant to dox.

### DNS-PE fluorescence

Dansyl-PE (0.4 mg) was dissolved in 10 μl ethanol and added to washed cell monolayers in 3 ml PBS ($10^6$ cells per 100 mm dish). Plates were shaken for 20 min at 25°C and subsequently washed four times with ice-cold PBS. Cells were then scraped into 1.5 ml of PBS and fluorescence intensity measured in a Perkin-Elmer MPF-44 Fluorescence Spectrophotometer. The excitation wavelength was 350 nm and emission wavelength was measured from 450–550 nm at a rate of 30 nm $min^{-1}$. Slit widths were set at 6 nm and samples were continuously stirred to provide a homogenous suspension. Fluorescence scans of dansyl-PE were also performed in a series of dioxane/water mixtures of known dielectric constant.

### TMA-DPH polarisation

Steady-state polarisation of TMA-DPH in whole cells was carried out according to previously published methods (Spiegel *et al.*, 1981). Measurements were made following a 10 min incubation (25°C) of $10^6$ cells in PBS containing 1 μM TMA-DPH. Polarisation was calculated according to the following equation:

$$P = (I_{vv} - I_{vh}.G)/(I_{vv} + I_{vh}.G)$$

where I is the emission intensity through polarisers orientated vertically ($I_{vv}$) and horizontally ($I_{vh}$) to the vertical plane of polarisation of the exciting light. G is the grating correction factor described by $I_{vh}/I_{hh}$. The wavelength of exciting light was 360 nm and the emission wavelength was set at 430 nm.

### TMA-DPH internalisation

The procedure used to monitor internalisation of TMA-DPH was based on published methods (Illinger *et al.*, 1990). Assays were performed on monolayers in 10 cm dishes ($10^6$ cells) and incubation times were 0,5, 10, 20 and 40 min. Following incubation, cells were washed with 3% fatty-free BSA in PBS to completely remove probe in the plasma membrane. Fluorescence intensity was measured with excitation and emission wavelengths set at 360 nm and 430 nm respectively.

### $^1H$ NMR analysis of cell membranes

Washed cell monolayers ($3 \times 10^7$ cells) were scraped into PBS and pelleted by centrifugation (200 *g*, 5 min). The pellet was frozen in liquid nitrogen and thawed in 4 ml chloroform:methanol (2:1). The suspension was briefly sonicated to disperse aggregates of cells and filtered through Whatman #1 paper. The filtrate was added to 50 mM NaCl (2 ml), mixed and centrifuged (3000 *g*, 5 min). The chloroform layer was evaporated and the residue suspended in 5 ml hexane. Anhydrous sodium sulphate was added to the hexane; the suspension mixed and centrifuged (3000 *g*, 5 min). Sodium sulphate was removed and hexane evaporated. The residue was dissolved in 500 μl deuterated chloroform and stored under argon at −20°C in 5 mm NMR tubes. The $^1H$ NMR spectra were recorded on a Bruker AM-500 spectrometer operating at a frequency of 500.13 MHz. Spectral parameters: sweep width 10 ppm, relaxation delay 3 s, spectral data size 16 K.

### Fatty acid analysis

Determination of the total fatty acid composition of cells was based on published methods (Rosenfeld *et al.*, 1986). Briefly, washed cell monolayers ($5 \times 10^7$ cells) were scraped into PBS and pelleted by centrifugation (2000 *g*, 5 min). The pellet was suspended in 4 ml $K_3PO_4$(pH 12.0) and boiled for 1 h at 100°C. The suspension was cooled and the pH adjusted to 7.4 with 1.0 M HCl. The neutralised suspension was then added to 300 mg of XAD-2 resin and 150 μl of 9:1 trichloroethylene:PFBBr. This mixture was shaken for 20 min at 25°C. The resin was separated and washed with distilled water. The derivitised fatty acids were eluted with 10 ml hexane. Water was removed with anhydrous sodium sulphate. Hexane was evaporated under nitrogen at 50°C and the residue dissolved in 400 μl of toluene containing 1 μg of external standard. Analyses were carried out on a Hewlett-Packard 5790 gas chromatograph with a Grob injector for splitless injection onto capillary columns and detected with a frequency pulsed electron capture detector. The capillary column was 30 m × 0.25 mm ID containing an SP330 phase (0.2 μm).

### Statistical analyses

All statistical analyses on comparisons of sample means were made using the unpaired Student's *t*-test. In all cases the 0.05 level was considered statistically significant.

### Results

### Dansyl-PE fluorescence maximum

The fluorescence emission of dansyl-PE in solution was sensitive to the dielectric constant of its surroundings (Ohki & Arnold, 1990). The emission peak of dansyl-PE was at a higher wavelength in the $CH^RC5$ cell line than in its drug sensitive parental line (Table I). An equivalent change in the

**Table I** The wavelength at maximal emission intensity of dansyl-phosphatidylethanolamine incorporated into various cell lines

| | Cell line | | | |
|---|---|---|---|---|
| | *AB1* | *$CH^RC5$* | *LR-73* | *LR-73-1A* |
| $\lambda_{em}$ (nm) | 509±1 | 517±1 | 513±2 | 514±1 |
| *P* | | <0.001 | | N.S. |

Values represent mean (±s.d.) of at least four experiments. *P* values indicate the significance in the difference in emission maximum between parental and resitant cell lines, i.e. AB1 *vs* $CH^RC5$ and LR-73 *vs* LR-73-1A.

emission peak for dansyl-PE in solution would suggest an increase in the surrounding dielectric constant of approximately 10 units. In contrast, there was no significant change in the emission peak of dansyl-PE in the transfected cell line (LR-73-1A) compare to its drug sensitive parental line (Table I).

*Steady-state fluorescence polarisation of TMA-DPH*

Steady-state polarisation measurements were performed on whole cells using the fluorescent probe TMA-DPH. The polarisation value was 14% higher in the CH$^R$C5 cell line compared to the parental cell line (Table II). Steady-state polarisation was also higher in the transfected cell line; however the magnitude of the change was higher (Table II).

*Membrane internalisation*

Cells were labelled with the fluorescent probe TMA-DPH and incubated at 37°C. Following incubation, the cells were washed with a BSA-PBS solution which removed >95% of the probe from the plasma membrane. The remaining fluorescence intensity was a measure of plasma membrane internalisation. Fluorescence intensities were expressed as a percentage of total intensity incorporated (i.e. in the absence of washing).

The rate of internalisation is initially linear with time and is 58% higher in the CH$^R$C5 cell line compared to its parental line (Table III). In addition, the mean final extent of internalisation was 20% higher in the CH$^R$C5 cells. The transfected cell line (LR-73-1A) also exhibited a greater initial rate of plasma membrane internalisation than its parental line; the magnitude of the difference was 108% (Table III). The mean final extent of internalisation was 51% higher in the LR-73-1A cell line.

The effect of ZfFG on plasma membrane internalisation was examined in the LR-73-1A cell line. Prior to incorporation of TMA-DPH, cell monolayers were incubated with 20 μM ZfFG at 37°C for 10 min. ZfFG reduced the mean initial rate of plasma membrane internalisation by 33% (Table IV). In addition, the extent of membrane internalisation was reduced by 21%.

*Cellular lipid composition determined by $^1$H-NMR*

A typical $^1$H-NMR spectrum obtained from a whole cell lipid extract is shown in Figure 1. Peak assignments were taken

**Table II** The steady-state polarisation of the fluorescent probe TMA-DPH in various cell lines

| | *Cell line* | | | |
|---|---|---|---|---|
| | *AB1* | *CH$^R$C5* | *LR-73* | *LR-73-1A* |
| Polarisation | 0.185±0.004 | 0.211±0.007 (+14%) | 0.173±0.009 | 0.215±0.007 (+24%) |
| *P* | | <0.02 | | <0.01 |

Values represent mean (±s.d.) of five independent experiments. *P* values indicate the significance in the difference in the polarisation between parental and resitant cell lines, i.e. AB1 *vs* CH$^R$C5 and LR-73 *vs* LR-73-1A.

**Table III** The rate and extent of TMA-DPH internalisation in various cell lines

| *Cell line* | *Rate of internalisation (% min$^{-1}$)* | *Extent of internalisation (%)* |
|---|---|---|
| AB1 | 0.67±0.02 | 30.9±1.9 |
| CH$^R$C5 | 1.06±0.05 | 37.1±1.1 |
| *P* | <0.001 | <0.01 |
| LR-73 | 0.50±0.02 | 15.7±1.3 |
| LR-73-1A | 1.04±0.07 | 23.7±2.5 |
| *P* | <0.001 | <0.01 |

Values are mean (±s.d.) of five independent experiments.

**Table IV** The effect of 20 μm ZfFG on the rate and extent of TMA-DPH internalisation in drug-resistant LR-73-1A cells

| *Concentration of ZfFG* | *Rate of internalisation (% min$^{-1}$)* | *Extent of internalisation (%)* |
|---|---|---|
| 0 | 0.91±0.07 | 19.7±1.0 |
| 20 μM | 0.61±0.07 | 15.6±1.1 |
| *P* | <0.01 | <0.02 |

Values represent the mean (± SD) of five independent experiments.

**Figure 1** Typical 500 MHz $^1$H-NMR spectra of whole cell (CH$^R$C5) extract. Peak assignment as follows: **a**, plasmalogen, **b**, sphingolipids, **c**, oleifinic (unsaturated lipids and cholesterol), **d**, glycerolipids, **e**, cholesterol esters, **f**, cholesterol, **g**, phosphatidylcholines, **h**, phosphatidylethanolamines, **i**, $\Delta^5$ unsaturated lipids, **j**, glycerol-fatty acid esters, **k**, methylene groups of acyl chains and **l**, terminal methyl group of cholesterol.

from published methods (Sze & Jardetzky, 1990). The relative amounts of total cholesterol esters, total phospholipid, phosphatidylcholine, phosphatidylethanolamine, $\Delta^5$ fatty acids, plasmolagens and sphingolipids were determined by integration of resonances unique to these lipid species. Phospholipid species such as phosphatidylinositol and phosphatidylserine were difficult to resolve but their intensities indicate they are minor components in each cell line.

$^{31}$P NMR spectra obtained from the above samples had a low signal-to-noise ratio and did not indicate the presence of phospholipids other than PE and PC (data not shown).

The CH$^R$C5 and LR-73-1A cell line did not differ from their parental lines with respect to composition of several lipid species (Table V). The PC:cholesterol ratio was approximately 1.0 in each cell line and the PC:PE ratio did not differ between the CH$^R$C5 and AB1 cells. The latter ratio was higher in the LR-73-1A cells but this difference was not statistically significant. The double bond index, a measure of unsaturation per phospholipid, was also invariant between the cell lines. This ratio suggests that approximately 58–68% of the fatty acid species were unsaturated. The amount of polyunsaturated lipids (i.e. the $\Delta^5$ fatty acids) was also similar in each cell line.

*Cellular composition of fatty acids*

A typical gas chromatogram of pentafluorobenzyl esters of fatty acids obtained from whole cell extracts is shown in

**Table V** The major lipid composition of whole cell extracts of various cell lines

| | *AB1* | *CH^R C5* | *LR-73* | *LR-73-1A* |
|---|---|---|---|---|
| TL | 46.5±5.8 | 49.1±3.8 | 52.4±3.5 | 50.4±2.7 |
| PC | 28.9±1.7 | 25.9±1.4 | 33.2±1.7 | 30.5±1.4 |
| PE | 13.6±1.8 | 12.5±1.3 | 14.1±1.7 | 11.5±2.1 |
| OL | 7.5±1.0 | 10.6±3.7 | 7.3±2.7 | 8.5±3.2 |
| ChL | 26.8±3.0 | 27.3±2.8 | 30.1±4.7 | 27.9±1.6 |
| ChL-E | 13.7±1.7 | 14.6±1.4 | 7.1±4.7 | 10.0±3.1 |
| PLN | 3.7±1.0 | 3.3±0.7 | 4.1±1.1 | 4.9±1.4 |
| SPH | 5.9±1.2 | 5.8±1.6 | 7.6±2.2 | 8.0±3.1 |
| PC/ChL | 1.11±0.17 | 0.96±0.13 | 1.11±0.15 | 1.09±0.07 |
| PC/PE | 2.12±0.18 | 2.08±0.12 | 2.39±0.23 | 2.72±0.48 |
| DBI | 1.23±0.09 | 1.16±0.10 | 1.35±0.17 | 1.34±0.32 |
| $\Delta^5$ index | 0.50±0.05 | 0.49±0.05 | 0.69±0.03 | 0.59±0.16 |

Proportion of each lipid species is expressed as a percentage of the total lipid determined. Values represent the mean (±s.d.) of five independent experiments. TL, total phospholipid (including di- and tri-glycerides); PC, phosphatidylcholine; PE, phosphatidylethanolamine; OL, other phospholipids; ChL, cholesterol; ChL-E, cholesterol-ester; PLN, plasmalogen; SPH, sphingolipids; DBI, double bond index. DBI and $\Delta^5$ represent the amount of double bounds and $\Delta^5$ groups per acyl chain.

**Table VI** The majority fatty acid species detected in various cell lines

| *Cell line* | *AB1* | *CH^R C5* | *LR-73* | *LR-73-1A* |
|---|---|---|---|---|
| 14:0 | 3.1±0.8 | 2.8±1.3 | 2.3±0.7 | 2.3±0.1 |
| 16:0 | 17.1±2.4 | 18.7±2.5 | 17.6±1.6 | 19.2±1.9 |
| 16:1 | 3.9±2.4 | 3.7±1.7 | 2.4±0.4 | 2.7±1.5 |
| 16:2 | 7.1±1.6 | 6.8±3.3 | 5.8±0.3 | 5.1±1.1 |
| 18:0 | 9.0±2.1 | 7.5±1.3 | 7.4±0.9 | 10.0±2.6 |
| 18:1 | 29.1±3.4 | 30.6±0.9 | 33.3±2.2 | 31.1±2.5 |
| 18:2 | 5.9±1.1 | 6.5±0.9 | 7.4±0.9 | 7.4±1.6 |
| 18:3 | 5.2±1.6 | 4.0±2.0 | 5.0±0.4 | 7.0±3.4 |
| 20:4 | 5.8±0.9 | 6.2±0.2 | 6.0±2.8 | 6.7±0.4 |
| 22:2 | 1.7±0.4 | 1.5±0.3 | 1.3±0.6 | 1.0±0.1 |
| 22:6 | 2.8±0.5 | 2.7±0.2 | 3.3±0.1 | 3.1±0.7 |
| Others | 9.8±3.7 | 5.7±2.2 | 7.8±4.4 | 3.1±1.2 |

Proportions of each fatty acid expressed as a percentage of the total fatty acid determined. Values represent the mean (±s.d.) of four independent experiments.

Figure 2. The major fatty acid species were identified from retention times of appropriate standards.

The relative amounts of the major fatty acid species (expressed as % of total fatty acid) did not differ significantly between the cell lines investigated (Table VI). The percentage of unsaturated fatty acids was between 68–71%, which concurs with data estimated by $^1$H-NMR. Polyunsaturated fatty acids comprised 26–31% of all fatty acids.

### Discussion

In this study the membrane biophysical properties of multidrug resistant cells were characterised. Drug resistant CH$^R$C5 cells did not differ from their parental line in total phospholipid or fatty acid composition. This makes less likely that there are differences in the lipids of the plasma membrane, but it does not eliminate this possibility. The physical properties of the plasma membranes of the resistant cells were however clearly different. The resistant CH$^R$C5 cells had a decreased surface hydrophobicity and fluidity and the cycling of their plasma membrane was higher. The CH$^R$C5 cells acquired resistance by clonal selection after long term exposure to the cytotoxic cholcicine (Ling & Thompson, 1974). Multiple members of the *mdr* gene family are expressed in clonally-selected multi-drug resistant cell lines (Gros *et al.*, 1991). Any or all of the *mdr* gene products may be directly or indirectly responsible for the alterations in the physical properties of the cell membrane, or the membrane changes may result from changes in the expression of other genes and hence may be specific for this particular clonally selected resistant cell line.

**Figure 2** Typical chromatogram of pentafluorobenzylesters of fatty acids obtained from whole cell (AB1) extracts (lower panel). A blank chromatogram is shown in the upper panel. Peak assignments as follows: **a**, $C_{14:0}$, **b**, $C_{16:0}$, **c**, $C_{16:1}$, **d**, $C_{16:2}$, **e**, $C_{18:0}$, **f**, $C_{18:1}$, **g**, $C_{18:2}$, **h**, $C_{18:3}$, **i**, $C_{20:4}$, **j**, $C_{22:2}$ and **k**, $C_{22:6}$.

Cells transfected with the *mdr* genes are less likely to have incurred changes in the expression of other genes. Recently, it was shown that retroviral transfer of the human *mdr*1 gene conferred a level of resistance in newly isolated cells which was proportional to P-glycoprotein density in the plasma membrane (Choi *et al.*, 1991). After culturing the transfected cells for 4 weeks, the level of resistance was still proportional to P-glycoprotein density, however the proportionality constant was different from that of the newly isolated cells. Thus for a particular cell condition, resistance is related to P-glycoprotein expression, but when comparing cells in different states it was shown that the level of resistance cannot be predicted solely on the basis of P-glycoprotein.

In this study we have examined membrane properties in a cell line transfected with the *mdr*1 gene (Schurr *et al.*, 1989) in which only the expression of this gene is increased. This cell line overexpresses the P-glycoprotein (Schurr *et al.*, 1989). No change in total lipid or fatty acid composition of this transfected cell line was observed in the present study. However, the polarisation of TMA-DPH fluorescence was increased. This indicates a decreased mobility of the acyl chains of membrane phospholipids and is similar to the change observed with the clonally selected CH$^R$C5 cell line. Thus, this change in membrane physical property appears closely associated with MDR although such changes in 'fluidity' are not observed in all MDR cell lines (Alton *et al.*, 1991).

The absence of any difference in lipid composition suggests that differences in the physical properties between parental and MDR cell lines arises from plasma membrane proteins such as the overexpression of the P-glycoprotein. However, this does not completely eliminate the possibility that the lipid environment may be different between the cell lines since the distribution of the fatty acid chains on the glycerol backbone of the lipid and the distribution among the lipid types was not determined nor was the subcellular distribution of lipid determined.

Unlike the CH$^R$C5 cell line, the transfected cells did not exhibit altered surface hydrophobicity. Selection for MDR results in amplification of several genes. Consequently, proteins other than P-glycoprotein are overexpressed (Meyers & Biedler, 1991). Some of these proteins, such as the EGF receptor, are localised in the plasma membrane. Insertion of any of these proteins or greater surface density of the P-glycoprotein may be responsible for the decrease in surface hydrophobicity of the CH$^{s}$C5 cell line.

The rate and extent of membrane cycling was also increased in both the clonally selected and the transfected MDR cells. Increased membrane traffic has been implicated as a mechanism of the resistance phenomenon (Beck, 1987). It has been proposed that cytotoxic agents may be concentrated in cytoplasmic vacuoles which subsequently fuse to the plasma membrane and extrude their contents. Membrane cycling is controlled by a wide variety of factor including membrane composition and protein-lipid interactions (Steinman *et al.*, 1983). We propose that the resistant cells have a less stable membrane (Boscoboinik & Epand, 1989) which results in increased membrane cycling. This is supported by the inhibition of membrane cycling by N-carbobenzoxy-D-Phe-L-Phe-Gly(ZfFG). ZfFG is a membrane stabilising peptide and has been shown to alter other membrane function such as cell fusion with viruses (Kelsey *et al.*, 1990), the non-bilayer phase transition temperature (Epand, 1986) and fusion of model membranes (Epand *et al.*, 1987).

In summary, this study has provided evidence of alterations in the membrane properties of drug-resistant cells. Some of these properties, such as the fluorescence polarisation of TMA-DPH and membrane cycling, are altered in both the selected CH$^{R}$C5 and the transfected cell lines.

We are grateful to Dr P. Gros, Department of Biochemistry, McGill University, Montreal and Dr V. Ling of the Ontario Cancer Institute for providing the cell line used. We are also grateful to Dr Jack Rosenfeld of the Department of Pathology, McMaster University for invaluable assistance in the fatty acid analysis. This work was supported by grants from the Medical Research Council of Canada (MA 7654) and the National Cancer Institute of Canada.

### References

ALON, N., BUSCHE, R., TUMMLER, B. & RIORDAN, J.R. (1991). In *Molecular and Cellular Biology of Multi-Drug Resistance in Tumor Cells*, Robinson, I. (ed.). pp.263–276. Plenum Publishing: N.Y.

ARSENAULT, L.A., LING, V. & KARTNER, N. (1988). Altered plasma membrane ultrastructure in multidrug-resistant cells. *Biochim, Biophys. Acta.*, **938**, 315–321.

BASRUR, V.S., CHITNIS, M.P. & MENON, R.S. (1985). Cell surface alterations in murine leukaemia P388 adriamycin resistant cells: studies on lectin-induced agglutination and rearrangement of lectin receptors. *Oncology*, **42**, 328–331.

BECK, W.T. (1987). The cell biology of multiple drug resistance. *Biochem. Pharm.*, **36**, 2879–2888.

BENCHEKROUN, M.N., VRIGNAUD, P., MONTAUDON, D. & ROBERT, J. (1988). Alteration of ganglioside composition and metabolism in doxorubicin-resistant rat tumoral cells. *Biochim. Biophys. Acta.*, **963**, 553–557.

BOSCOBOINIK, D. & EPAND, R.M. (1989). Increased cellular internalization of amphiphiles in a multidrug-resistant CHO cell line. *Biochim. Biophys. Acta.*, **1014**, 53–56.

CHOI, K., FROMMEL, T.O., STERN, R.K., PEREZ, C.F., KRIEGLER, M., TSURUO, T. & RONINSON, I.G. (1991). Multidrug resistance after retroviral transfer of the human MDR1 gene correlates with P-glycoprotein density in the plasma membrane and is not affected by cytotoxic selection. *Proc. Natl Acad. Sci. USA*, **88**, 7386–7390.

EPAND, R.M. (1986). Virus replication inhibitory peptide inhibits the conversion of phospholipid bilayers to the hexagonal phase. *Bioscience Rep.*, **6**, 647–653.

EPAND, R.M. & LEON, T.-C. (1992). Hexagonal phase forming propensity detected in phospholipid bilayers with fluorescent probes. *Biochemistry*, **31**, 1550–1554.

EPAND, R.M., EPAND, R.F. & MCKENZIE, R.C. (1987). Effects of viral chemotherapeutic agents on membrane properties: Studies of cyclosporin A carbobenzoxy-D-Phe-L-Phe-Gly and amantadine. *J. Biol. Chem.*, **262**, 1526–1529.

ESCRIBA, P.V., FERRER-MONTIEL, A.V., FERRAGUT, J.A. & GONZALEZ-ROS, J.M. (1990). Role of membrane lipids in the interaction of daunomycin with plasma membranes from tumor cells: Implications in drug-resistance phenomena. *Biochemistry*, **29**, 7275–7282.

GOTTESMAN, M.M. & PASTAN, I. (1988). Resistance to multiple chemotherapeutic agents in human cancer cells. *TIPS*, **9**, 54–58.

GROS, P., BEN NERIAH, Y., CROOP, J. & HOUSEMAN, D.E. (1986). Isolation and expression of a complementary DNA that confers multidrug resistance. *Nature*, **323**, 728–731.

GROS, P., RAYMOND, M. & HOUSEMAN, D.E. (1991). In *Molecular and Cellular Biology of Multi-Drug Resistance in Tumor Cells*, Roninson, I. (ed.). pp.73–89. Plenum Publishing: N.Y.

ILLINGER, D., POINDRON, P., FONTENEAU, P., MODOHEL, M. & KUHRY, J.G. (1990). Internalization of the lipophilic fluorescent probe trimethylamino-diphenylhexatriene follows endocytosis and recycling of the plasma membrane on cells. *Biochim. Biophys. Acta.*, **1030**, 73–81.

JULIANO, R.L. & LING, V. (1976). A surface glycoprotein modulating drug permeability in Chinese hamster ovary cell mutants. *Biochim. Biophys. Acta.*, **455**, 152–162.

KELSEY, D.R., FLANAGAN, T.D., YOUNG, J. & YEAGLE, P.L. (1990). Peptide inhibitors of enveloped virus infection inhibit phospholipid vesicle fusion and Sendai virus fusion with phospholipid vesicles. *J. Biol. Chem.*, **265**, 12178–12183.

KESSEL, D. (1988). Probing membrane alterations associated with anthracycline resistance using fluorescent dyes. *Biochem. Pharmacol.*, **37**, 4253–4256.

LING, V. & THOMPSON, L.H. (1974). Reduced permeability in CHO cells as a mechanism of resistance to colchicine. *J. Cell Physiol.*, **83**, 103–116.

MEYERS, M.B. & BIEDLER, J.L. (1991). In *Molecular and Cellular Biology of Multi-Drug Resistance in Tumor Cells*, Roninson, I. (ed.). pp.243–262. Plenum Publishing: N.Y.

OHKI, S. & ARNOLD, K. (1990). Surface dielectric constant, surface hydrophobicity and membrane fusion. *J. Membrane Biol.*, **114**, 195–203.

PETERSON, R.H.F., MEYERS, M.B., SPENGLER, B.A. & BIEDLER, J.L. (1983). Alteration of plasma membrane glycopeptides and gangliosides of Chinese hamster cells accompanying development of resistance to daunorubicin and vincristine. *Cancer Res.*, **43**, 222–228.

POWIS, G., HICKMAN, J., WORKMAN, P., TRITTON, T.R., ABITA, J.-P., BERDEL, W.E., GESCHER, A., MOSES, H.L. & NICOLSON, G.L. (1990). The cell membrane and cell signals as targets in cancer chemotherapy. *Cancer Res.*, **50**, 2203–2211.

RAMU, A., GLAUBINGER, D. & WEINTRAUB, H. (1984). Differences in lipid composition of doxorubicin-sensitive and doxorubicin-resistant P388 cells. *Cancer Treat. Rep.*, **68**, 637–641.

RIORDAN, J.R., DEUCHARS, K., KARTNER, N., ALON, N., TRENT, J. & LING, V. (1985). Amplification of P-glycoprotein genes in multidrug resistant mammalian cell lines. *Nature*, **316**, 817–819.

ROSENFELD, J.R., HAMMERBERG, O. & OVIDAS, M.C. (1986). Simplified methods for preparation of microbial fatty acids for analysis by gas-chromatography with electron capture-detection. *J. Chrom.*, **378**, 9–16.

SCHURR, E., RAYMOND, M., BELL, J.C. & GROS, P. (1989). Characterization of the multidrug resistant protein expressed in cell clones stably transfected with the mouse *mdr1* cDNA. *Cancer Res.*, **49**, 2729–2734.

SEHESTED, M., SKOVSGAARD, T., VAN DEURS, B. & WINTHER-NIELSON, H. (1987*a*). Nonspecific adsortive endocytosis in anthracycline-resistant and vinca alkaloid-resistant Ehrlich ascites tumor-cell lines. *J. Natl Cancer Inst.*, **78**, 171–177.

SEHESTED, M., SKOVSGAARD, T., VAN DEURS, B. & WINTHER-NIELSEN, H. (1987*b*). Increased plasma membrane traffic in daunorubicin resistant P388 leukaemic cells. Effect of daunorubicin and verapamil. *Br. J. Cancer*, **56**, 747–751.

SEHESTED, M., BINDSLEV, N., DEMANT, E.J.F., SKOVSGAARD, T. & JENSEN, P.B. (1989). Daunorubicin and vincristine binding to plasma membrane vesicles from daunorubicin-resistant and wild type Ehrlich ascites tumor cells. *Biochem. Pharmacol.*, **38**, 3017–3027.

SORANZO, C., INGROSSO, A., BUFFA, M., DELLA TORRE, G., GAMBETTA, R.A. & ZUNINO, F. (1989). Changes in the three-dimensional organization of LoVo cells associated with resistance to doxorubicin. *Cancer Lett.*, **48**, 37–41.

SPIEGEL, K.J., MAGRATH, I.T. & SHUTTA, J.A. (1981). Role of cytoplasmic lipids in altering diphenylhexatriene fluorescence polarization in malignant cells. *Cancer Res.*, **41**, 452–458.

STEINMAN, R.M., MELLMAN, I.S., MULLER, W.A. & COHN, Z.A. (1983). Endocytosis and the recycling of plasma membrane. *J. Cell Biol.*, **96**, 1–27.

SZE, D.Y. & JARDETZKY, O. (1990). Characterization of lipid composition in stimulated human lymphocytes $^{1}$H-NMR. *Biochim. Biophys. Acta.*, **1054**, 198–206.

TAPIERO, H., MISHAL, Z., WIOLAND, M., SILBER, A., FOURCADE, A. & ZWINGELSTEIN, G. (1986). Changes in biophysical parameters and in phospholipid composition associated with resistance to doxorubicin. *Anticancer Res.*, **6**, 649–652.

VRIGNAUD, P. & ROBERT, J. (1987). Free fatty acid uptake is increased in doxorubicin-resistant at glioblastoma cells. *Biochim. Biophys. Acta.*, **902**, 149–153.

WHEELER, C., RADER, R. & KESSEL, D. (1982). Membrane alterations associated with progressive adriamycin resistance. *Biochem. Pharmacol.*, **31**, 2691–2693.

WILDER, P.J., OVERMAN, D.K., TENENHOLZ, T.C. & GUTIERREZ, P.L. (1990). Differences in myristic acid synthesis and in metabolic rate for P388 cells resistant to doxorubicin. *J. Lipid Res.*, **31**, 1973–1982.

WRIGHT, L.C., DYNE, M., HOLMES, K.T. & MOUNTFORD, C.E. (1985). Phospholipid and ether linked phospholipid content alter with cellular resistance to vinblastine. *Biochem. Biophys. Res. Commun.*, **133**, 539–545.